# A cluster-randomized, non-inferiority trial comparing use of misoprostol for universal prophylaxis vs. secondary prevention of postpartum hemorrhage among community level births in Egypt

**DOI:** 10.1186/s12884-020-03008-5

**Published:** 2020-05-24

**Authors:** Holly A. Anger, Rasha Dabash, Nevine Hassanein, Emad Darwish, Mohamed Cherine Ramadan, Medhat Nawar, Dyanna Charles, Miral Breebaart, Beverly Winikoff

**Affiliations:** 1 Gynuity Health Projects, 220 E 42nd St, Suite 710, New York, NY USA; 2 Independent Reproductive Health Consultant, Cairo, Egypt; 3 Faculty of Medicine, Alexandria University, 17 Champollion St, El Messalah, Alexandria, Egypt; 4 Department of Obstetrics and Gynecology, El Galaa Teaching Hospital, Cairo, Egypt; 5 El Beheira Governorate, Ministry of Health and Population, Damanhour, Egypt; 6 Independent Public Health Consultant, Cairo, Egypt

**Keywords:** Misoprostol, Postpartum hemorrhage, Low and middle income countries, Secondary prevention, Prophylaxis, Home-births, Midwives, Task-sharing

## Abstract

**Background:**

Previous community-based research shows that secondary prevention of postpartum hemorrhage (PPH) with misoprostol only given to women with above-average measured blood loss produces similar clinical outcomes compared to routine administration of misoprostol for prevention of PPH. Given the difficulty of routinely measuring blood loss for all deliveries, more operational models of secondary prevention are needed.

**Methods:**

This cluster-randomized, non-inferiority trial included women giving birth with nurse-midwives at home or in Primary Health Units (PHUs) in rural Egypt. Two PPH management approaches were compared: 1) 600mcg oral misoprostol given to all women after delivery (i.e. primary prevention, current standard of care); 2) 800mcg sublingual misoprostol given only to women with 350-500 ml postpartum blood loss estimated using an underpad (i.e. secondary prevention). The primary outcome was mean change in pre- and post-delivery hemoglobin. Secondary outcomes included hemoglobin ≥2 g/dL and other PPH interventions.

**Results:**

Misoprostol was administered after delivery to 100% (1555/1555) and 10.7% (117/1099) of women in primary and secondary prevention clusters, respectively. The mean drop in pre- to post-delivery hemoglobin was 0.37 (SD: 0.91) and 0.45 (SD: 0.76) among women in primary and secondary prevention clusters, respectively (difference adjusted for clustering = 0.01, one-sided 95% CI: < 0.27, *p* = 0.535). There were no statistically significant differences in secondary outcomes, including hemoglobin drop ≥2 g/dL, PPH diagnosis, transfer to higher level, or other interventions.

**Conclusions:**

Misoprostol for secondary prevention of PPH is comparable to universal prophylaxis and can be implemented using local materials, such as underpads.

**Trial registration:**

Clinicaltrials.gov NCT02226588, date of registration 27 August 2014.

## Background

Administration of a uterotonic agent such as misoprostol as secondary prevention is an innovative approach to management of postpartum hemorrhage (PPH), the leading global cause of maternal morbidity and mortality [1–3]. In contrast to a universal prophylaxis strategy in which all delivering women receive a uterotonic after giving birth, the secondary prevention strategy (i.e. early treatment) features selective administration of a uterotonic given only to women who experience above-average bleeding but are not yet clinically emergent. While universal prophylaxis with a uterotonic is shown to reduce postpartum bleeding by about 79 ml (95% confidence interval [CI]: 62–96 ml) [4], evidence suggests that such routine use may be unnecessary for many women, particularly those deemed at “low risk” of bleeding [4, 5]. Use of a practical secondary prevention approach may alleviate the perceived need to medicate 100% of women after giving birth. Further, for women delivering at lower levels of care where access to emergency obstetric care is limited and where providers are often not authorized to treat PPH, a secondary prevention strategy may promote more active postpartum monitoring and facilitate a quicker response with first-line intervention to women with incipient PPH.

Evidence from a cluster-randomized trial of community births in India shows that administration of 800 mcg misoprostol (the recommended dose for PPH treatment [6, 7]) to only 5% of women as secondary prevention is clinically non-inferior to universal administration of 600 mcg misoprostol as prophylaxis for PPH [2]. Women in secondary prevention clusters also had fewer side effects. Misoprostol is a safe, effective option for prevention and treatment of PPH in settings where use of oxytocin, the gold standard uterotonic for PPH, is not feasible due to its parenteral administration and refrigeration requirements [8–13]. Use of misoprostol for secondary prevention is an attractive option for programs in need of sustainable solutions with more focus on postpartum monitoring and that go beyond point-in-time interventions. This strategy may be particularly important at lower levels of care where resources are scarce and where timely access to PPH treatment is not always feasible.

In the India trial, administration of misoprostol for secondary prevention was triggered by blood loss ≥350 ml (an amount previously shown to represent the lower limit of the top quartile of women with measured postpartum blood loss [14, 15]) as measured with a blood collection drape. Though a useful research tool, the blood collection drape is not widely available and is unlikely to be used on a large scale. Further, it is questionable whether such precision in blood loss measurement is needed. Secondary prevention is likely as effective when less exact tools are used to aid providers to recognize when blood loss is more than normal. For secondary prevention to become an operational reality, evidence is needed on a more practical tool to aid a birth attendant in monitoring blood loss and triggering administration of secondary prevention.

To this aim, the present study investigates a more operational model of triggering secondary prevention of PPH with misoprostol with an inexpensive, locally available absorbent underpad that is often used during or after delivery in Egypt and many other countries as a protective layer beneath women. We tested the hypothesis that this more operational model of secondary prevention was no worse than routine administration of misoprostol as universal prophylaxis.

## Methods

This parallel, cluster-randomized, non-inferiority trial took place between November 2015 and January 2016 among deliveries attended by nurse-midwives in the rural districts of Kafr el Dawar and Damanhour, El Beheira governorate, Egypt. Nurse-midwives are affiliated with Primary Health Units (PHUs) in the district and attend deliveries occurring at women’s homes and PHUs. Nurse-midwives were randomized to administer misoprostol as universal prophylaxis or as secondary prevention among deliveries they attended during the study period; randomization was performed according to the PHU where the midwife worked and the deliveries conducted by nurse-midwives affiliated with each PHU formed the clusters. PHUs were considered for inclusion in this study if nurse-midwives at the PHU attended an average of at least 25 deliveries a month. Before the start of enrollment, the 21 PHUs (representing 32 nurse-midwives) were stratified by district and by volume of deliveries (categorized as low, medium, and high by tertiles) and were then randomized to the universal prophylaxis or secondary prevention arms using a 1:1 allocation ratio. Gynuity Health Projects performed the randomization using a computer-generated sequence applied in each stratum. The unit of randomization was the PHU in order to prevent contamination among nurse-midwives working at the same PHU who otherwise could be randomized to different arms. There was no masking because this would have made it impossible to assess key service delivery aspects of the two strategies, such as feasibility and acceptability.

During the study period, women were screened for eligibility by nurse-midwives during the 3rd trimester antenatal care visit or during early labor. Eligibility criteria included: plan to give birth at home or a PHU with a nurse-midwife from a participating PHU; agree to pre and post-delivery assessment of hemoglobin; agree to follow-up interview; no known allergy to misoprostol or prostaglandins. Women with pregnancy complications (such as hypertension, suspected multiple pregnancy, previous caesarean section, suspected stillbirth, ante-partum hemorrhage, and previous complication in the third trimester) were often instructed to deliver at the hospital and were thus rarely included in the study unless they still opted to deliver at home. Informed consent was documented by the woman’s signature or thumbprint. At the time of enrollment, nurse-midwives measured pre-delivery hemoglobin via a portable handheld device (Hemocue, Angelhom, Sweden). Information on demographics, obstetric history and the delivery were collected by nurse-midwives using standardized data collection instruments.

Nurse-midwives provided standard of care during the second stage of labor. Pads were provided to nurse-midwives to aid in assessment of postpartum blood loss. These locally available underpads measure 60 cm × 90 cm, cost $0.05 USD, and absorb approximately 350–500 ml blood when folded in half. Prior to study launch, the study team assessed the absorption capacity of the underpad by applying known quantities of blood, and then by using the pad during deliveries to monitor postpartum blood loss and weighing the soaked blood pad 1 h after delivery to estimate the quantity of blood absorbed. These initial assessments confirmed that women with blood loss that came near to saturating the underpad had blood loss (determined objectively by wieghing the underpad and subtracting the known wieght of the pad) of 350–500 ml. Nurse-midwives in both study arms were instructed to fold the mat in half, place it under all women after giving birth, and to monitor the blood loss for at least 1 h.

Nurse-midwives randomized to the universal prophylaxis arm administered 600 mcg oral misoprostol (three 200 mcg tablets) immediately after birth. Those randomized to the secondary prevention arm administered 800 mcg sublingual misoprostol (four 200 mcg tablets under the tongue for 20 min) only to women with postpartum blood loss that soaked the folded underpad and/or had other early signs of PPH (e.g. change in vital signs, relaxed uterus). Information on misoprostol administration, observed blood loss, occurrence of side effects (including shivering, fever, nausea, vomiting, diarrhea), and immediate postpartum care was recorded by nurse-midwives after delivery.

In both study arms, immediate referral to higher level care was advised if blood loss continued after the pad was soaked. PPH diagnosis could also be made based on other factors per individual clinical judgement according to factors such as change in vital signs and the woman’s general condition. Nurse-midwives performed follow-up visits to all women at their homes 2–4 days after delivery to measure their postpartum hemoglobin levels and to administer a brief exit interview about the woman’s experience with the care provided.

The primary outcome for this study was mean change in hemoglobin pre- and post-delivery. Secondary outcomes included a drop in pre- to post-delivery hemoglobin ≥2 g/dL, PPH diagnosis, transfer to higher level care due to PPH, administration of additional interventions to control PPH (such as additional uterotonics, bimanual compression, etc.), occurrence of side effects, acceptability of care received (including tolerability of any side effects), and programmatic feasibility. PPH was diagnosed according to the birth attendant’s clinical judgement, and could be based on continued bleeding observed after soaking the pad, rate of blood loss, or other clinical factors (i.e. change in vital signs).

Our a priori non-inferiority hypothesis stated that secondary prevention would be considered clinically non-inferior to primary prevention if the mean change in pre- to post-delivery hemoglobin observed in secondary prevention clusters was ≤0.3 g/dL compared to the mean hemoglobin change observed in primary prevention clusters. After assuming a pooled standard deviation of 1.1 g/dL, the unadjusted sample size estimate was 334 deliveries (one-sided α = 0.05, 80% power), and after considering an intracluster correlation (ICC) of 0.05 and average cluster size of 135, we estimated that 2400 deliveries were needed to account for the cluster design effect [16]. Per these parameters, the null hypothesis (i.e. non-inferiority of secondary prevention) would be rejected if the one-sided 95% confidence interval (CI) for difference in mean change in hemoglobin fell below 0.3 g/dL.

Risk ratios and associated 95% CIs were calculated using log-binomial regression models for categorical outcomes and mean differences in continuous outcomes were estimated by calculating regression coefficients and 95% CIs via linear regression models. Generalized estimating equations (GEE) was used for all regression models to account for clustering by PHU. A one-sided *p* value and 95% CI was calculated to test the non-inferiority hypothesis; all other *p* values and 95% CIs were two-sided. Analyses were done using an intent-to-treat approach, so all women were included in the final analysis, unless they later became ineligible after enrollment (e.g. transferred and gave birth at a hospital rather than at home or the PHU). All analyses were performed using Stata Version 12.0 (StataCorp. 2011. *Stata Statistical Software: Release 12*. College Station, TX: StataCorp LP).

This study is registered with Clinicaltrials.gov (#NCT02226588).

## Results

**Fig. 1:**
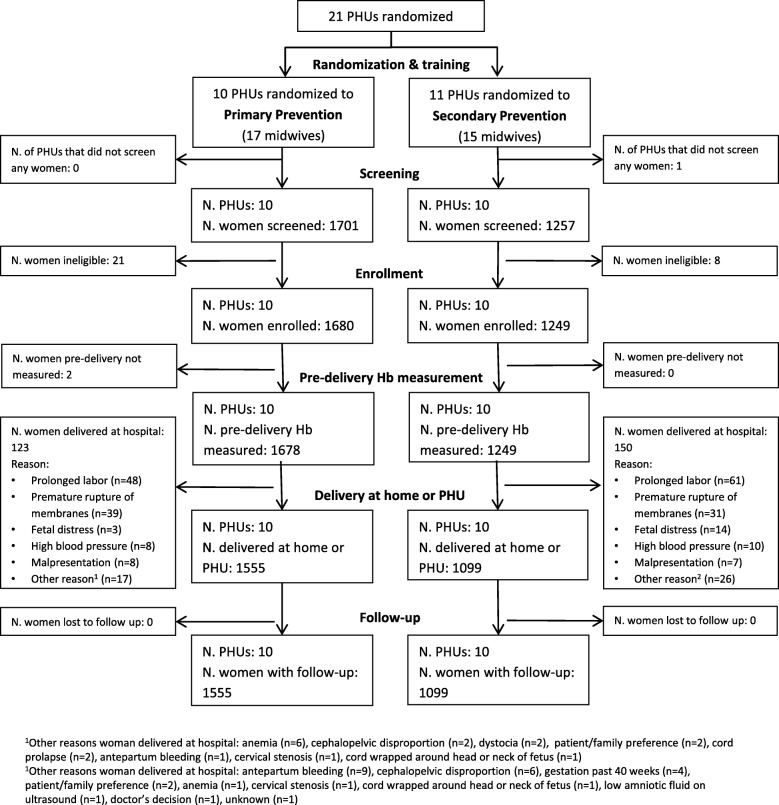
Study flowchart of cluster-randomized, non-inferiority trial. Abbreviations: PHU=Primary Health Unit, Hb = hemoglobin, ^1^Other reasons women delivered at hospital: anemia (*n* = 6), cephalopelvic disproportion (*n* = 2), dystocia (*n* = 2), patient/family preference (*n* = 2), cord prolapse (*n* = 2), antepartum bleeding (*n* = 1), cervical stenosis (*n* = 1), cord wrapped around head or neck of fetus (*n* = 1), ^2^Other reasons women delivered at hospital: antepartum bleeding (*n* = 9), cephalopelvic disproportion (*n* = 6), gestation past 40 weeks (*n* = 4), patient/family preference (*n* = 2), anemia (*n* = 1), cervical stenosis (*n* = 1), cord wrapped around head or neck of fetus (*n* = 1), low amniotic fluid on ultrasound (*n* = 1), doctor’s decision (*n* = 1), unknown (*n* = 1)

Before the study commenced, 10 and 11 PHUs were randomized to the primary prevention and secondary prevention strategies, respectively (Fig. 1). All sites except one in the secondary prevention arm screened women for eligibility in the study. There were 1680 and 1249 women enrolled in primary and secondary prevention clusters, respectively. Some enrolled women later delivered at the hospital and were thus excluded from the analysis because they became ineligible, including 123/1680 (7.3%) and 150/1249 (12.0%) in primary and secondary prevention clusters, respectively (Fig. 1). Most women who delivered at the hospital did so because they experienced labor complications, such as prolonged labor or premature rupture of membranes. The final analysis of non-inferiority included 1555 women in primary clusters and and 1099 women secondary prevention clusters. The study groups were similar with respect to demographics and obstetric history characteristics (Table 1). Most women delivered at the woman’s or midwife’s home (99.0 and 99.8% in primary and secondary prevention clusters, respectively). Compared to women in primary prevention clusters, use of uterotonics for labor induction or augmentation was higher in secondary prevention clusters (0.6% vs 3.8%); oxytocin was the main uterotonic used before birth in both primary prevention (9/10) and secondary prevention clusters (27/42), although misoprostol was also used (used in 1/10 and 18/42 women in primary and secondary prevention clusters, respectively, who received a uterotonic before delivery). Several women in both study groups received a uterotonic (other than the study misoprostol) during the third stage of labor (0.1 and 1.0% in primary and secondary prevention arms, respectively). The underpad was used for almost all women in both study arms (99.7% in primary prevention and 100% in secondary prevention clusters). As these births all occurred in homes or PHUs, there were no births by cesarean section or assisted vaginal births.

**Table 1:** Demographics and delivery characteristics of women enrolled in the study

	N	Primary Prevention	Secondary Prevention
N clusters	20	10	10
N of women enrolled	2654	1555	1099
N women per cluster, average (range)	20	156 (48–317)	110 (35–188)
**Demographics and Obstetric History**^a^			
Age, mean (SD)	2653	25.4 (5.0)	25.8 (5.1)
Education^b^			
No/informal education	726	408 (26.3%)	318 (29.1%)
Primary/Preparatory	795	505 (32.6%)	290 (26.5%)
Secondary and above	1124	638 (41.1%)	486 (44.4%)
Nulliparous	562	348 (22.4%)	214 (19.5%)
Gravida, mean (SD)	2654	2.4 (1.1)	2.5 (1.1)
Known PPH in past pregnancies^c^	48	25 (1.6%)	23 (2.1%)
**Delivery Characteristics**^a^			
Place of birth			
Home of woman or midwife	2636	1539 (99.0%)	1097 (99.8%)
PHU	18	16 (1.0%)	2 (0.2%)
Pre-delivery Hb,			
Mean (SD)	2654	11.3 (1.2)	11.3 (1.1)
Pre-delivery Hb < 11.0	1015	595 (38.3%)	420 (38.2%)
Gestational age when Hb measured in weeks, mean (SD)	2654	38.7 (1.3)	38.9 (1.3)
Uterotonic given before delivery	52	10 (0.6%)	42 (3.8%)
Procedure during 3rd stage of labor			
Uterine massage	1139	709 (45.6%)	430 (39.1%)
Controlled cord traction	357	222 (14.3%)	135 (12.3%)
Non-study uterotonic given during 3rd stage of labor	2654	2 (0.1%)	11 (1.0%)
Blood absorption mat used after delivery	2649	1550 (99.7%)	1099 (100%)

^a^Results are presented as N (%) except where otherwise noted

^b^4 women in PP group and 5 women in SP group had unknown education level

^c^37 women in PP group and 4 women in SP group had unknown information for previous PPH

All women (1555/1555, 100%) giving birth in primary prevention clusters received the study intervention of 600mcg oral misoprostol during the third stage of labor and 117/1099 (10.7%) women in secondary prevention clusters received 800mcg sublingual misoprostol for secondary prevention. More women in secondary prevention clusters had blood loss that soaked the mat (10.2% vs. 0.6% in primary prevention clusters). All women in secondary prevention clusters with blood loss that soaked the mat received 800 mcg misoprostol as secondary prevention. Among 117 women who received misoprostol for secondary prevention, the most common reason was because postpartum bleeding soaked the mat. Only 5/117 women who received the secondary prevention intervention had blood loss that did not soak the mat; nurse-midwives said they chose to administer misoprostol to these women due to concerns over vital signs, rate of blood loss, retained placenta, and/or atonic uterus.

### Non-inferiority test

**Fig. 2:**
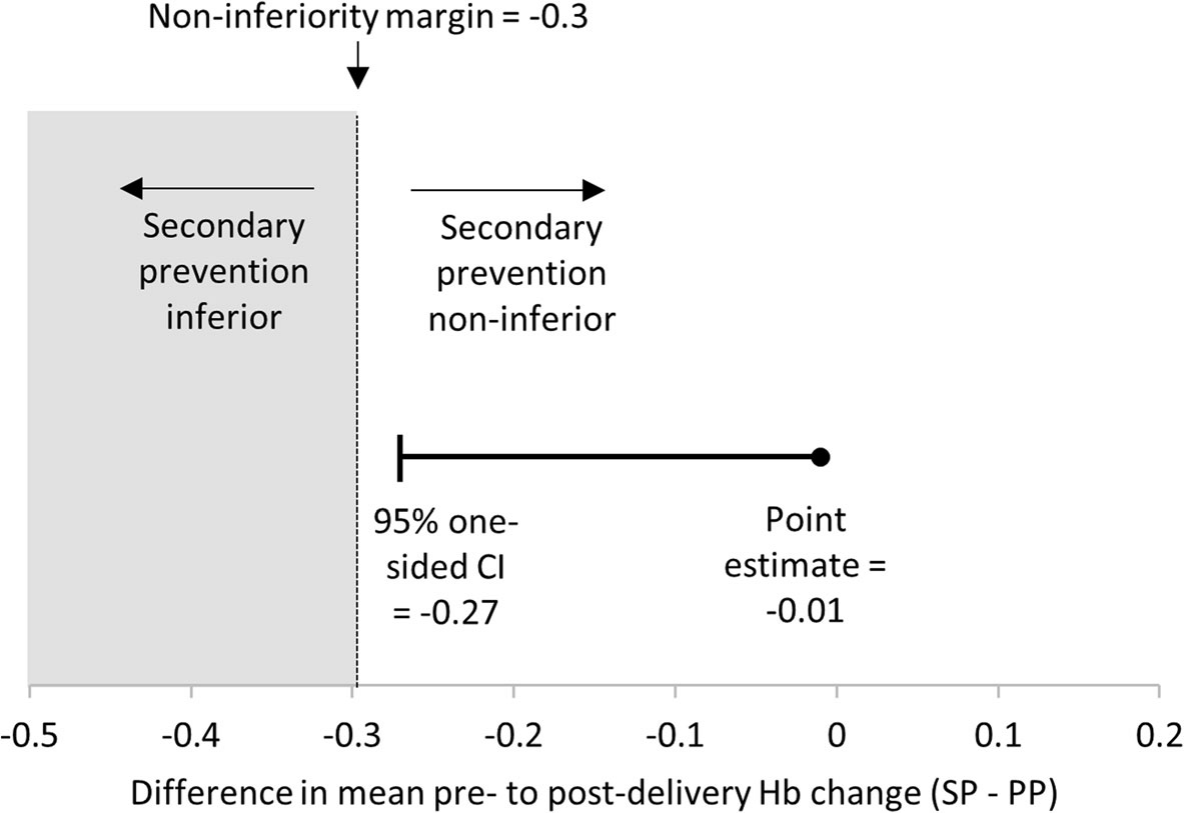
Non-inferiority test. Both the point estimate and one 95% confidence interval for change in pre- and post-delivery hemoglobin falls above the a priori-defined non-inferiority margin of -0.3, indicating that secondary prevention is non-inferior to primary prevention. Abbreviations: CI=Confidence interval, Hb = hemoglobin, SP = secondary prevention, PP = primary prevention

Among the total 2654 women with pre- and post-delivery hemoglobin measurement, the mean change in pre- to post-delivery hemoglobin was − 0.37 (SD: 0.91) and − 0.45 (SD: 0.76) among women in primary and secondary prevention clusters, respectively (Table 2). The adjusted estimate for difference in mean change in hemoglobin showed that women in secondary prevention clusters had a mean hemoglobin drop that was 0.01 g/dL larger than the drop experienced by women in primary prevention clusters. The one-sided 95% CI was 0.27 for this difference, indicating that women in secondary prevention were not likely to have experienced a hemoglobin drop that was 0.3 g/dL larger (the pre-defined non-inferiority margin) than the hemoglobin drop observed among women in primary prevention clusters (Fig. 2).

**Table 2:** Outcomes among women delivering in primary and secondary prevention clusters

	N	Primary Prevention^**1,2**^	Secondary Prevention^**1,3**^	ICC	Estimate^**4**^	95% CI	***P*** value^**5**^
**Non-inferiority test**							
Hb change	2654	*N* = 1555	*N* = 1099				
Mean (SD)		−0.37 (0.91)	−0.45 (0.76)	0.16	β = -0.01	Not less than -0.27^6^	0.535
**Secondary outcomes**							
Hb drop ≥2 g/dL	2654	*N* = 1555	*N* = 1099				
		81 (5.2%)	28 (2.6%)	0.07	RR = 0.46	0.15, 1.37	0.161
Post-delivery Hb	2654	*N* = 1555	*N* = 1099				
Mean (SD)		10.9 (1.2)	10.8 (1.2)	0.10	β = 0.08	−0.26, 0.42	0.616
PPH diagnosis	2654	*N* = 1555	*N* = 1093				
		5 (0.3%)	6 (0.6%)	0.02	RR = 2.17	0.28, 16.46	0.455
Transfer to higher level care	2654	*N* = 1555	*N* = 1099				
		2 (0.1%)	1 (0.1%)	< 0.01	RR = 0.77	0.09, 6.46	0.810
Additional uterotonics^7^	2654	*N* = 1555	*N* = 1099				
		4 (0.3%)	2 (0.2%)	0.01	RR = 0.52	0.05, 5.15	0.573

^1^Results are presented as N (%) except where otherwise noted

^2^All 1555 in primary prevention clusters received 600mcg oral misoprostol; 9/1555 (0.6%) women in primary prevention clusters soaked the underpad

^3^112/1099 (10.2%) women in secondary prevention clusters soaked the underpad; 117 (10.7%) women in secondary prevention clusers received 800mcg misoprostol, including all 112 who soaked the underpad

^4^*β* Regression coefficient derived incorporating generalized estimating equations (GEE), *RR* Risk ratio derived from log-binomial regression incorporating generalized estimating equations (GEE)

^5^*P* value for Hb change is one-sided, all other *p* values are two-sided

^6^One sided 95% CI for non-inferiority test, pre-defined non-inferiority margin = -0.3

^7^Additional uterotonics administered at place of birth or at the hospital if transferred

### Secondary outcomes

Drops in hemoglobin of ≥2 g/dL were less common among women in secondary prevention clusters compared to women in primary prevention clusters (2.6% vs. 5.2%, RR: 0.46), though this difference was not statistically significant (95% CI: 0.15, 1.37) (Table 2). Similarly, there was no significant difference in mean post-delivery hemoglobin between the two groups. There were no statistically significant differences in PPH diagnosis, transfer to higher level care, or use of additional uterotonics, all which were rare in both study groups (< 1%). There were no cases of maternal death, surgical intervention for PPH, or blood transfusion in either study arm.

### Side effects and acceptability

Compared to women delivering in primary prevention clusters, fewer women in secondary prevention clusters experienced shivering (42.6% vs. 27.8%, respectively) and vomiting (10.1% vs 4.2%, respectively), though these differences were not statistically significant (RR for shivering: 0.58, 95% CI 0.27–1.24, RR for vomiting 0.53, 95% CI: 0.18–1.55) (Table 3). Feeling faint was more common among women in the secondary prevention arm (RR: 10.54, 95% CI: 2.34–47.46), though it was a rare occurrence in both study groups (0.1 and 1.4% in primary and secondary prevention clusters, respectively). The study groups were comparable with respect to other side effects and their severity.

**Table 3:** Side effects reported among women delivering in primary and secondary prevention clusters

	Primary Prevention^**a**^ ***N*** = 1555	Secondary Prevention^**a**^ ***N*** = 1099	ICC	Risk ratio^**b**^	95% CI	***P*** value
**Shivering**	**663 (42.6%)**	**305 (27.8%)**	**0.33**	**0.58**	**0.27–1.24**	**0.158**
Severe	23 (1.5%)	17 (1.6%)				
**Fever**	**6 (0.4%)**	**2 (0.2%)**	**< 0.01**	**0.56**	**0.06–5.25**	**0.614**
Severe	0	0				
**Diarrhea**	**3 (0.2%)**	**1 (0.1%)**	**< 0.01**	**0.61**	**0.05–8.20**	**0.708**
Severe	0	0				
**Nausea**	**48 (3.1%)**	**41 (3.7%)**	**0.01**	**1.24**	**0.60–2.53**	**0.561**
Severe	2 (0.1%)	2 (0.2%)				
**Vomiting**	**157 (10.1%)**	**46 (4.2%)**	**0.15**	**0.53**	**0.18–1.55**	**0.249**
Severe	1 (0.1%)	0				
**Fainting/feel faint**	**2 (0.1%)**	**15 (1.4%)**	**0.01**	**10.54**	**2.34–47.46**	**0.002**
Severe	1 (0.1%)	0				
**Other**^**c**^	**22 (1.4%)**	**5 (0.5%)**	**0.09**	**0.28**	**0.03–2.29**	**0.236**
Severe	2 (0.1%)	0				

^a^Results are presented as N (%)

^**b**^Risk ratio derived from log-binomial regression incorporating generalized estimating equations (GEE)

^c^Other side effects in Group 1: abdominal pain/cramping (*n* = 18), pain in nipple (*n* = 1), numbness (*n* = 2), headache (*n* = 1) and in Group 2: fatigue (*n* = 1), cold sweats and low blood pressure (*n* = 1), chest pain (*n* = 1), feeling faint (*n* = 1) and high blood pressure (*n* = 1)

Acceptability of both primary and secondary prevention protocols was high among women. Among women who took misoprostol in primary and secondary prevention clusters, 1546/1555 (99.4%) and 115/115 (100%), respectively, said that they would take it again (data was missing for 2 women who received misoprostol in secondary prevention clusters). When asked if they would be willing to pay for misoprostol, 99.5% of women overall said they would be willing to pay for its use as primary prevention and 92.4% would be willing to pay for its use as secondary prevention.

## Discussion

The results of this community trial confirm findings from a previous study in India showing that use of misoprostol for secondary prevention of PPH is clinically non-inferior to use of misoprostol as universal prophylaxis [2]. The two studies together show that, with active monitoring of blood loss, a secondary prevention model that medicates just 5–11% of women is no worse than a universal approach that medicates 100% of women. Compared to the India trial, this study presents a more operational model of secondary prevention, as it shows that use of a simple, imprecise tool such as a blood pad (i.e. bed underpad or “chux” pads found in many settings) can effectively aid providers in determining an appropriate point to administer secondary prevention. While more women in secondary prevention clusters had blood loss that soaked the pad, this amount did not result in higher rates of hemoglobin drop ≥2 g/dL, PPH diagnosis, transfers to higher level care, or other PPH interventions. These findings corroborate conclusions from prior systematic literature reviews showing that, while universal prophylaxis may reduce postpartum blood loss, there is no evidence that these reductions result in decreases in maternal morbidity or mortality [17, 18]. These two studies on secondary prevention add to a growing body of evidence suggesting that routine administration of uterotonics may not be necessary following vaginal birth, particularly among “low risk” births [4, 19], and these studies go one step further to suggest that this conclusion may apply in low and middle income settings.

There are several reasons why secondary prevention may be preferred over universal prophylaxis. First, fewer women would be medicated and experience associated side effects. Though our study did not show a statistically significant reduction in side effects among women delivering in the secondary prevention arm, the rate of shivering was markedly lower (28% vs. 43%), and the India study did show a significant reduction in shivering [2]. Second, secondary prevention has a cost savings advantage, since not all delivering women would incur the cost of the medication as would occur with the universal prophylaxis approach, and these savings could be considerable when distributed over a whole population [20]. Further, in low and middle-income settings where stockouts in facilities are common, this strategy may provide a more resourceful use of commodities such as uterotonics. Finally, secondary prevention may empower birth attendants such as nurse-midwives in Egypt to play an active role in PPH management, and this may facilitate more timely diagnosis and initial treatment. Like many community midlevel birth attendants throughout the world, nurse-midwives in Egypt are not authorized to treat PPH. These birth attendants are expected to diagnose PPH and refer such women to higher levels, but they often lack the proper tools and training to provide first-line treatment. Given the very low rates of transfer of women diagnosed with PPH in this study, it is clear that immediate first-line interventions such as uterotonics should be considered essential wherever women deliver.

There is an urgent need for broader thinking about the role of birth attendants in PPH management and for improvements in care provision in the immediate postpartum period, as it is the time women are most at risk of PPH and is also “the most neglected period for the provision of quality care,” as stated by the World Health Organization [21]. One advantage of universal prophylaxis may be that it is a simpler approach that could reduce errors compared to secondary prevention, as universal prophylaxis entails administration to all women without the requirement to monitor and assess blood less. On the other hand, use of universal prophylaxis may instill a false sense of security for some birth attendants, as they may feel they have “prevented” PPH after administering prophylaxis and that the job is done. Prior studies of facility-based births show that there can be relatively widespread use of prophylactic uterotonics and concurrent low rates of adequate postpartum monitoring of women [22–24]. This could be risky, as studies show that some women who receive a prophylactic uterotonic will still go on to have PPH [8, 9, 25]. Compared to universal prophylaxis, a secondary prevention approach may better focus the birth attendant’s attention to monitoring the woman’s condition during the immediate postpartum period, as this is necessary to determine if the secondary prevention dose of a uterotonic is needed. We were unable to examine this phenomenon in this study, as the study protocol instructed nurse-midwives in both primary and secondary prevention arms to actively monitor and record blood loss for 1 h postpartum; this was done because our main research question was whether selectively medicating some women with above-average bleeding was no worse than medicating 100% of women. It is unclear if use of a secondary prevention model could result in improvements in postpartum monitoring and if so, whether this would translate to improvements in maternal health outcomes. While our research focused on assessing the use of misoprostol for secondary prevention in a community setting, future facility-based research may explore use of secondary prevention with oxytocin in low and middle income settings, where postpartum monitoring is often sub-standard and where there is growing concern with overuse of uterotonics. Additional research would help further elucidate the comparative benefits of secondary prevention and better define the population best suited for this care model.

Rates of PPH morbidity in this study population were low, both in terms of diagnosed PPH and hemoglobin drops ≥2 g/dL. Our study enrolled women giving vaginal birth at home or at PHUs, thus women with pregnancy complications and risk factors for excessive bleeding were often excluded in our study, as they were encouraged to have a hospital birth. Yet, it has also been shown that a large proportion of women diagnosed with PPH have no known risk factors [26], thus it is plausible that our study population still reflects a large proportion of women that may go on to experience severe PPH. Nevertheless, it is uncertain if the findings indicating non-inferiority of secondary prevention would be generalizable to populations with higher rates of PPH morbidity. It is also notable that the rates of hemoglobin drop ≥2 g/dL were higher than rates of diagnosed PPH in both our study groups. This may be explained by additional blood loss that occurred after the study’s one-hour monitoring period when study providers documented rates of diagnosed PPH; the hemoglobin drop would reflect total postpartum blood loss, not just that observed in the 1 h postpartum. One previous study also noted higher rates of pre- to post-delivery hemoglobin drops ≥2 g/dL in comparison to diagnosed PPH, and the authors similarly hypothesized that the discrepancy may be explained by persistent or discontinuous bleeding that occurred after the close monitoring period immediately after the birth [27]. Hemoglobin drops ≥2 g/dL were also slightly more common among women in primary prevention clusters (5.2%) than in secondary prevention clusters (2.6%), even though only 0.6% of women in primary prevention clusters had blood loss that soaked the underpad compared to 10.2% of women in secondary prevention clusters. In the secondary prevention arm, women with blood loss that soaked the underpad received the regimen of 800 mcg sublingual misoprostol, and prior trials have shown that 89–90% of women with PPH have their active bleeding controlled within 20 min of receiving this misoprostol regimen [14, 15]. Hence, administration of 800 mcg sublingual misoprostol among women with above-average bleeding in secondary prevention clusters may have worked to quickly stimulate uterine contractions and stop bleeding, thereby preventing large drops in hemoglobin.

A limitation of our study is the uneven enrollment between primary and secondary prevention clusters, despite having stratified clusters by reported delivery volume prior to randomization. This phenomenon in not uncommon in cluster-randomized trials [28], and careful study monitoring ensured that these differences were due to chance and not to any preference or selection practices on the part of women or providers. We also ensured that the harmonic mean of the group sample sizes (*n* = 1287) was above 1200 women, the required number by sample size calculations. Another limitation is that the randomization for this study was done by Primary Health Unit, which could introduce bias if the populations differed greatly by Primary Health Unit; however, comparison of baseline characteristics by primary and secondary prevention clusters did not show important differences that would impact study outcomes, and we employed appropriate statistical methods (i.e. generalized estimating equations) to adjust our analysis for clustering. Our study is strengthened by the systematic measurement of both pre- and post-delivery hemoglobin, which allowed us to clearly compare the physiological effect of postpartum blood loss among women in the two groups. Regular study monitoring ensured strict compliance with the study protocol, ensuring that the models of care were implemented as intended.

## Conclusion

This study provides important evidence on the comparative safety and effectiveness of use of misoprostol as secondary prevention for PPH management. As do their counterparts in high resource settings, women in low and middle-income countries deserve high quality maternity care that entails appropriate and timely use of interventions. Further, birth attendants in low- and middle-income settings require training and models of care that go beyond administering prophylaxis and that give them the tools and knowledge to appropriately monitor for, diagnose, and treat abnormal postpartum bleeding (and to transfer when necessary). A secondary prevention approach provides a promising alternative to universally medicating every woman that delivers in a community setting, and it is an important opportunity to expand access to PPH management to wherever women choose to deliver.

## Data Availability

The data that support the findings of this study are available from the corresponding author upon reasonable request.

## Abbreviations

PPH: Postpartum hemorrhage
PHU: Primary health unit
ICC: Intracluster correlation coefficient
GEE: Generalized estimating eqs.
RR: Risk ratio
CI: Confidence interval
SD: Standard deviation
